# Magnesium at the Neurovascular Interface: A Narrative Review of Atherosclerosis, Peripheral Arterial Disease, and Neuropathic Pain

**DOI:** 10.3390/nu18111675

**Published:** 2026-05-23

**Authors:** Yonghyun Yoon, Rowook Park, Jaehyun Shim, Junyoung Park, Jihyo Hwang, Jungyoun Kim, King Hei Stanley Lam, Teinny Suryadi, Anwar Suhaimi

**Affiliations:** 1 Department of Orthopaedic Surgery, Gangnam Sacred Heart Hospital, College of Medicine, Hallym University, 1 Singil-ro, Yeongdeungpo-gu, Seoul 07441, Republic of Korea; 2 Incheon Terminal Orthopedic Surgery Clinic, Inha-ro 489beon-gil, Namdong-gu, Incheon 21574, Republic of Korea; 3 International Academy of Regenerative Medicine, Inha-ro 489beon-gil, Namdong-gu, Incheon 21574, Republic of Korea; 4 The Board of Clinical Research, International Academy of Musculoskeletal Medicine, Kowloon, Hong Kong; painfreedoc22@gmail.com (T.S.);; 5 Department of Rehabilitation Medicine, Sae Yonsei Rehabilitation Clinic, Seoul 03186, Republic of Korea; 6 Department of Neurosurgery, Chungdammadi Neurosurgery Clinic, Seoul 03186, Republic of Korea; 7 Faculty of Medicine, The University of Hong Kong, Kowloon, Hong Kong; 8 Faculty of Medicine, The Chinese University of Hong Kong, New Territory, Hong Kong; 9 Department of Physical Medicine and Rehabilitation, Medistra Hospital, South Jakarta 12950, Indonesia; 10 Department of Physical Medicine and Rehabilitation, Synergy Clinic, West Jakarta 11470, Indonesia; 11 Department of Rehabilitation Medicine, Universiti Malaya, Kuala Lumpur 50603, Malaysia

**Keywords:** magnesium, atherosclerosis, endothelial dysfunction, neuropathic pain, ischemic pain, NMDA receptor, neuroinflammation, vascular calcification, diabetic neuropathy, pain sensitization

## Abstract

Magnesium (Mg) is an essential divalent cation involved in more than 600 enzymatic reactions and plays a fundamental role in vascular, metabolic, and neural homeostasis. Although Mg is frequently discussed as an analgesic supplement, emerging evidence suggests that it acts as a neurovascular–metabolic modulator. Low magnesium status has been associated with endothelial dysfunction, atherosclerotic burden, impaired microcirculatory function, and overlapping ischemic and neuropathic pain phenotypes, although direct causal clinical evidence remains limited. This narrative review integrates mechanistic and clinical evidence across three intersecting domains: (1) the role of Mg in endothelial dysfunction, vascular calcification, and atherogenesis; (2) the contribution of Mg deficiency to ischemic pain through peripheral arterial disease and microcirculatory failure; and (3) the modulation of neuropathic pain through NMDA receptor antagonism, neuroinflammatory suppression, and maintenance of blood–brain barrier integrity. In populations with atherosclerosis, diabetes mellitus, or nutritional insufficiency, hypomagnesemia may serve as a unifying pathophysiological link connecting vascular injury to pain sensitization. The recognition of Mg not merely as an analgesic agent, but as a neurovascular interface regulator, may inform more comprehensive therapeutic strategies in chronic vascular and neuropathic pain syndromes. This review emphasizes nutritional magnesium status and biologically plausible mechanisms rather than presenting magnesium supplementation as an established treatment for vascular or neuropathic pain. The evidence is strongest for mechanistic vascular and neuropathic pathways, whereas direct clinical evidence for magnesium supplementation in PAD-related ischemic limb pain remains limited.

## 1. Introduction

Magnesium (Mg) is the fourth most abundant mineral in the human body and the second most prevalent intracellular cation after potassium, functioning as a cofactor in over 600 enzymatic systems governing energy metabolism, nucleic acid synthesis, ion channel regulation, and neurotransmitter release [1,2]. A significant proportion of adults consume less than the recommended dietary allowance, which generally ranges from 310 to 420 mg/day depending on age and sex [3]. This latent deficiency state, often subclinical and undetected by routine serum measurements, carries substantial consequences for cardiovascular and neurological health [4].

The traditional clinical framing of Mg has been predominantly pharmacological, focusing on its use as a tocolytic, antiepileptic, or adjuvant analgesic administered intravenously in acute settings. However, this perspective fails to capture the broader biological significance of chronic, low-grade Mg deficiency as a contributor to atherosclerotic and neuropathic disease trajectories. Epidemiological data consistently demonstrate that low dietary Mg intake and low serum Mg are independently associated with hypertension, coronary artery calcification, peripheral arterial disease (PAD), stroke, and cardiovascular mortality [4,5]. Mechanistically, Mg deficiency promotes endothelial dysfunction, oxidative stress, vascular smooth muscle cell transdifferentiation, and neuroinflammation, pathophysiological processes that converge at the interface between vascular disease and pain [1,6].

Recent public health-oriented reviews have further emphasized that suboptimal magnesium status remains common across populations and is influenced by dietary patterns, chronic disease, medication use, and lifestyle-related factors, supporting the need to interpret magnesium deficiency not only as an individual biochemical abnormality but also as a broader nutritional and public health issue [7].

Pain in the context of atherosclerosis and its complications is heterogeneous in origin. Ischemic pain arising from impaired microcirculation in peripheral arterial disease represents a distinct phenotype from neuropathic pain complicating diabetic or ischemic neuropathy, yet both may share a common upstream driver in Mg deficiency. In patients with type 2 diabetes, a well-documented vicious cycle exists wherein hypomagnesemia worsens insulin resistance, which in turn reduces renal Mg reabsorption, further depleting systemic Mg stores [8]. This cycle simultaneously damages both the vasculature and peripheral nerves, creating the substrate for co-occurring ischemic and neuropathic pain.

This narrative review seeks to reframe Mg within a neurovascular–metabolic paradigm, examining its roles across three interconnected domains: endothelial biology and atherogenesis, peripheral vascular ischemia and microcirculatory pain, and neuropathic pain sensitization through central and peripheral neuromodulatory mechanisms. Rather than treating these as separate topics, we propose that low Mg status may represent an integrating pathophysiological factor linking vascular injury to pain phenotypes in metabolically vulnerable populations.

### Review Scope and Literature Selection

This narrative review was designed to synthesize mechanistic and clinical literature relevant to magnesium, vascular dysfunction, peripheral arterial disease, ischemic pain, and neuropathic pain. Literature searches were conducted in PubMed, Google Scholar, and the reference lists of relevant reviews and primary studies from database inception to May 2026. To improve transparency while maintaining the narrative-review design, the search process was organized into five topic-specific blocks: magnesium and vascular dysfunction/atherosclerosis; magnesium and peripheral arterial disease/ischemic pain; magnesium and neuropathic pain/NMDA receptor signaling; magnesium and neuroinflammation/blood–brain barrier integrity; and magnesium and diabetes, metabolic syndrome, or neuropathy.

The searches identified 1044 records across these five topic-specific blocks: magnesium and vascular dysfunction/atherosclerosis, 211 records; magnesium and peripheral arterial disease/ischemic pain, 7 records; magnesium and neuropathic pain/NMDA receptor signaling, 113 records; magnesium and neuroinflammation/blood–brain barrier integrity, 65 records; and magnesium and diabetes, metabolic syndrome, or neuropathy, 648 records. After removal of 78 duplicate records, 966 unique records were screened for relevance.

Eligible sources included peer-reviewed mechanistic studies, animal studies, observational studies, randomized clinical trials, systematic reviews, narrative reviews, clinical guidelines, and major nutritional reference sources addressing magnesium status, magnesium supplementation, vascular dysfunction, peripheral arterial disease, ischemic or neuropathic pain, or diabetes-related neurovascular complications. Studies were excluded if they were non-peer-reviewed conference abstracts, duplicate publications, focused on clinical contexts outside the scope of this review, did not primarily address magnesium or the target neurovascular outcomes, or lacked accessible methodological or outcome information. Based on these criteria and the conceptual scope of this narrative review, the most relevant mechanistic, translational, epidemiological, clinical, guideline, and nutritional reference sources were incorporated into the final narrative synthesis. Ultimately, 64 sources were included in the final review.

**Figure 1:**
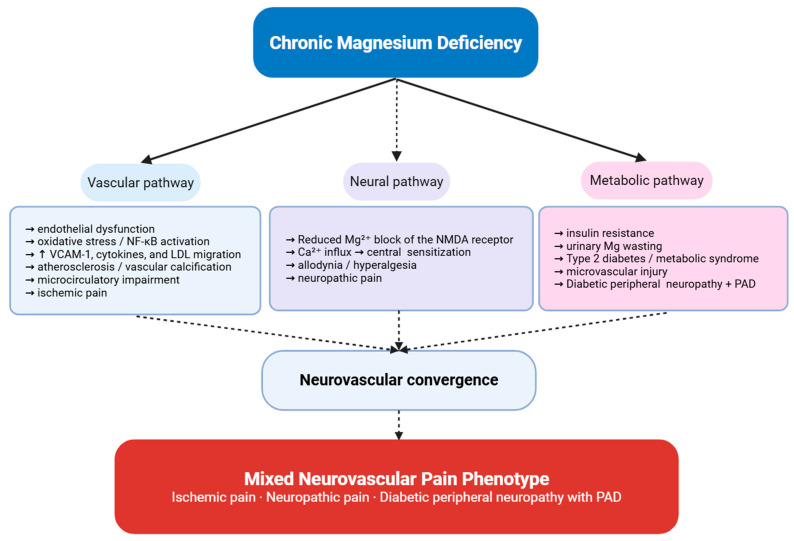
Proposed integrative framework linking chronic magnesium deficiency to mixed neurovascular pain phenotypes. Chronic magnesium deficiency may contribute to neurovascular pain through three interconnected pathways: a vascular pathway, a neural pathway, and a metabolic pathway. In the vascular pathway, magnesium deficiency promotes endothelial dysfunction, oxidative stress, inflammatory activation, atherosclerosis, vascular calcification, and microcirculatory impairment, ultimately contributing to ischemic pain. In the neural pathway, reduced Mg^2+^-dependent blockade of the NMDA receptor facilitates Ca^2+^ influx, central sensitization, allodynia, hyperalgesia, and neuropathic pain. In the metabolic pathway, magnesium deficiency is linked to insulin resistance, urinary magnesium wasting, type 2 diabetes, metabolic syndrome, and microvascular injury, thereby contributing to diabetic peripheral neuropathy and peripheral arterial disease (PAD). These pathways converge at the neurovascular interface and may manifest clinically as mixed neurovascular pain phenotypes, including ischemic pain, neuropathic pain, and diabetic peripheral neuropathy with PAD. In the figure, upward arrows (↑) denote increased activity, expression, or progression of the adjacent biological process, mediator, or clinical consequence. Evidence grading is indicated by arrow style and relative thickness: thick solid arrows represent links supported by human clinical or epidemiological evidence, whereas thinner dashed arrows represent mechanisms or integrative links supported mainly by preclinical, mechanistic, or clinically unconfirmed evidence. Abbreviations: Mg, magnesium; Ca, calcium; NMDA, N-methyl-D-aspartate; NF-κB, nuclear factor-kappa B; VCAM-1, vascular cell adhesion molecule-1; LDL, low-density lipoprotein; PAD, peripheral arterial disease.

This review does not aim to provide a comprehensive overview of all magnesium-related cardiovascular, metabolic, neurological, or pain conditions. Instead, it focuses on selected evidence relevant to the neurovascular consequences of low magnesium status and its potential contribution to overlapping ischemic and neuropathic pain phenotypes. Because the purpose of this article is conceptual integration rather than quantitative evidence synthesis, no formal meta-analysis was performed. Accordingly, this review should be interpreted as a narrative synthesis of mechanistic, translational, epidemiological, and clinical evidence rather than as a systematic review. An overview of the proposed framework linking chronic magnesium deficiency to vascular dysfunction, neural sensitization, metabolic dysregulation, and mixed neurovascular pain phenotypes is presented in Figure 1.

## 2. Magnesium Homeostasis and Assessment

Total body Mg content in adults is approximately 24 g, of which approximately 60% resides in bone, 39% in muscle and soft tissues, and only about 1% in the extracellular compartment, including the plasma [3]. This distribution explains why serum Mg, the most commonly measured clinical parameter, is an unreliable indicator of total body Mg status. Serum Mg concentrations are tightly maintained within a reference range of 0.7–1.0 mmol/L through a coordinated balance of intestinal absorption, bone exchange, and renal excretion; deficits in intracellular or bone Mg can persist despite normal serum values [3,9].

The principal transporters governing cellular Mg homeostasis are the transient receptor potential melastatin channels (TRPM)6 and TRPM7 [8]. TRPM6 is predominantly expressed in the kidney and intestinal epithelium and is the primary determinant of urinary Mg reabsorption, whereas TRPM7 is ubiquitously expressed and maintains intracellular Mg homeostasis in virtually all cell types, including vascular endothelial cells, vascular smooth muscle cells (VSMCs), and neurons [1,10]. Insulin directly activates renal TRPM6, and in insulin-resistant states such as type 2 diabetes, impaired TRPM6 activity leads to increased urinary Mg wasting and progressive hypomagnesemia [8]. Thus, insulin resistance itself becomes a proximate cause of Mg deficiency, and the resulting hypomagnesemia in turn exacerbates insulin resistance, creating a self-amplifying pathological cycle [8,11].

Assessment of Mg status remains clinically challenging. Serum total Mg may be within the normal range despite significant intracellular depletion, a phenomenon sometimes termed “normomagnesemia masking.” More sensitive assessment approaches include erythrocyte Mg content, intracellular ionized Mg measured by nuclear magnetic resonance, and the 24 h urinary Mg excretion test, though these remain largely limited to research settings [9]. This limitation is consistent with recent reviews emphasizing that no single routinely available biomarker fully captures total-body or intracellular magnesium status, and that improved diagnostic strategies are needed for both clinical and public health applications [7]. Practically, clinicians should maintain a high index of suspicion for functional Mg deficiency in populations at risk, including patients with poorly controlled diabetes, chronic kidney disease, use of proton pump inhibitors or loop diuretics, alcohol dependence, or chronically suboptimal dietary intake [1,12]. Because serum total magnesium may remain within the normal range despite intracellular depletion, magnesium status should be interpreted using both laboratory data and clinical context. In addition to serum total Mg and intracellular or urinary measures, the serum calcium-to-magnesium ratio has recently been proposed as a potential complementary tool for identifying adverse magnesium/calcium imbalance. However, this approach remains emerging and should be interpreted as an adjunctive assessment rather than a validated replacement for conventional Mg evaluation [13,14]. The main methods for assessing magnesium status and relevant clinical risk factors are summarized in Table 1.

## 3. Magnesium and Endothelial Function: The Gate to Atherosclerosis

### 3.1. Endothelial Dysfunction Under Magnesium Deficiency

The endothelium constitutes a dynamic biological interface between the bloodstream and the vascular wall, orchestrating vasomotor tone, inflammatory signaling, coagulation, and barrier permeability. Endothelial dysfunction, characterized by reduced nitric oxide (NO) bioavailability, increased expression of adhesion molecules, and enhanced permeability, is recognized as the initiating step of atherogenesis [6]. A growing body of experimental and clinical evidence positions Mg deficiency as a potent inducer of endothelial dysfunction [6,15].

In vitro studies using human umbilical vein endothelial cells (HUVECs) and human microvascular endothelial cells cultured in low-Mg media have consistently demonstrated a pro-inflammatory, pro-atherogenic phenotypic shift [15,16]. Specifically, low extracellular Mg increases NADPH oxidase activity, upregulates thioredoxin-interacting protein (TXNIP), and generates excessive reactive oxygen species (ROS), which in turn activate nuclear factor-kappa B (NF-κB), the master transcriptional regulator of inflammation [15]. This cascade culminates in the upregulation of inducible nitric oxide synthase (iNOS), paradoxically generating excess NO through an inflammatory rather than vasodilatory pathway, thereby disrupting endothelial barrier function [15]. Simultaneously, Mg deficiency suppresses endothelial NO synthase (eNOS) expression, reduces prostacyclin (PGI_2_) synthesis, and elevates endothelin-1 (ET-1), a potent vasoconstrictor, creating a state of sustained endothelial vasoconstrictor dominance [1].

The inflammatory phenotype induced by low Mg is further characterized by increased secretion of interleukin-1α (IL-1α), IL-6, IL-8, and tumor necrosis factor-α (TNF-α), enhanced surface expression of vascular cell adhesion molecule-1 (VCAM-1), and increased adhesion of monocytoid cells to the endothelium [16,17]. These events facilitate the recruitment of circulating monocytes and their differentiation into macrophages within the subendothelial space, a pivotal early step in atherosclerotic plaque formation. Low Mg also upregulates matrix metalloproteinases (MMP-2 and MMP-9) while the inhibitory effect of tissue inhibitor of metalloproteinase-2 (TIMP-2) is overridden, resulting in accelerated degradation of extracellular matrix proteins, increased endothelial permeability, and enhanced transendothelial migration of low-density lipoproteins (LDL) [1].

An important and relatively novel mechanism linking Mg deficiency to atherogenesis is the accumulation of lipid droplets within endothelial cells. Mg deficiency upregulates the endothelial differentiation factor EDF-1 and peroxisome proliferator-activated receptor-γ (PPARγ) through oxidative stress-dependent pathways, leading to intracellular lipid deposition, an early atherogenic event within the vascular wall [18]. Additionally, Mg deficiency induces features of premature cellular senescence in endothelial cells, including upregulation of the cyclin-dependent kinase inhibitor p21 and increased beta-galactosidase activity, processes that further contribute to chronic endothelial dysfunction and vascular aging [17].

Clinical evidence corroborates these mechanistic observations. A randomized, double-blind, placebo-controlled trial in 50 stable coronary artery disease (CAD) patients demonstrated that 6 months of oral Mg supplementation (30 mmol/day) significantly improved brachial artery flow-mediated dilatation (FMD), a validated measure of endothelium-dependent vasodilation, by 15.5% compared with baseline, with a significant correlation between baseline intracellular Mg concentrations and baseline FMD [19]. In hemodialysis patients, oral Mg supplementation (440 mg MgO three times per week for 6 months) significantly decreased carotid intima-media thickness (cIMT), a structural marker of subclinical atherosclerosis [20].

### 3.2. Magnesium Deficiency and Vascular Calcification

Vascular calcification, defined as the ectopic deposition of calcium-phosphate crystals in the arterial wall, represents a critical mechanism through which atherosclerosis translates into arterial stiffness, impaired microcirculation, and cardiovascular events [21]. Mg inhibits vascular calcification through two complementary mechanisms: passive physicochemical interference with hydroxyapatite crystal formation in the extracellular space, and active cellular modulation of VSMC transdifferentiation toward an osteogenic phenotype [21,22].

**Figure 2:**
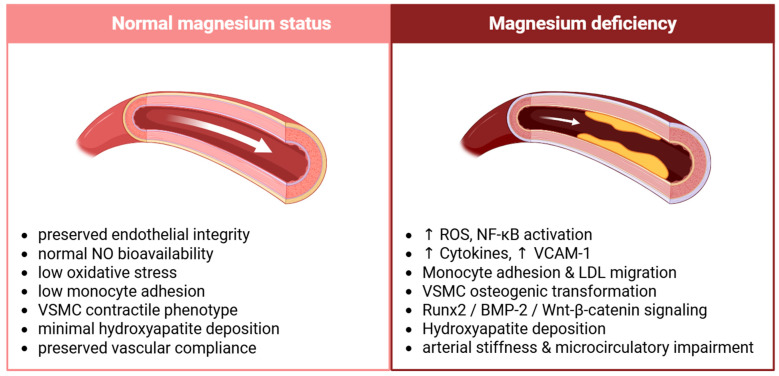
Comparison of vascular features under normal magnesium status and magnesium deficiency. Under normal magnesium status, vascular homeostasis is maintained through preserved endothelial integrity, normal nitric oxide (NO) bioavailability, low oxidative stress, reduced monocyte adhesion, preservation of the contractile phenotype of vascular smooth muscle cells (VSMCs), minimal hydroxyapatite deposition, and preserved vascular compliance. In contrast, magnesium deficiency promotes oxidative stress and NF-κB activation, increases cytokine production and VCAM-1 expression, enhances monocyte adhesion and LDL transendothelial migration, and induces osteogenic transformation of VSMCs through pathways involving Runx2, BMP-2, and Wnt/β-catenin signaling. These changes facilitate hydroxyapatite deposition, arterial stiffness, and microcirculatory impairment, thereby contributing to vascular dysfunction and atherosclerotic progression. Arrows indicate the proposed direction of pathophysiological progression or mechanistic linkage between the depicted vascular processes; they are schematic and do not denote quantified effect size. Abbreviations: Mg, magnesium; NO, nitric oxide; NF-κB, nuclear factor-kappa B; VCAM-1, vascular cell adhesion molecule-1; LDL, low-density lipoprotein; VSMCs, vascular smooth muscle cells; BMP, bone morphogenetic protein.

In the extracellular compartment, Mg ions compete with calcium for binding sites on nascent hydroxyapatite crystals, altering crystal lattice composition and reducing crystal growth rates [22]. The passive buffering of phosphate by Mg reduces the supersaturation of calcium-phosphate in the vessel wall, effectively delaying calcification initiation [23]. In the cellular compartment, VSMCs exposed to high-phosphate environments undergo osteoblastic transdifferentiation, upregulating the master osteogenic transcription factors Runx2/Cbfa-1, osterix, and bone morphogenetic proteins (BMP-2, BMP-4) while downregulating calcification inhibitors such as matrix Gla protein (MGP) and osteopontin (OPN) [10]. Magnesium, by activating TRPM7 channels in VSMCs and inhibiting the Wnt/β-catenin signaling pathway, a key driver of osteogenic differentiation, prevents this phenotypic transition and restores the expression of anti-calcification proteins [24]. Accordingly, the present review focuses on peer-reviewed evidence supporting passive physicochemical inhibition of hydroxyapatite formation and active modulation of VSMC osteogenic transdifferentiation through TRPM7- and Wnt/β-catenin-related pathways. The clinical relevance of Mg in vascular calcification is most clearly illustrated in chronic kidney disease (CKD), where impaired Mg handling co-occurs with severe vascular calcification. Observational data in dialysis patients consistently show that higher serum Mg concentrations are associated with reduced prevalence and progression of vascular calcification and improved survival [23,25]. In a prospective study, lower serum Mg was independently associated with an almost 2-fold higher hazard ratio for incident PAD (HR: 1.96; 95% CI: 1.40–2.74), supporting the pathophysiological link between hypomagnesemia, calcification, and peripheral ischemic disease [26]. The vascular consequences of magnesium deficiency, including endothelial dysfunction, inflammatory activation, VSMC osteogenic transformation, and hydroxyapatite deposition, are summarized in Figure 2.

### 3.3. Oxidative Stress, Dyslipidemia, and the Inflammatory Cascade

Mg deficiency generates a pro-oxidant milieu through multiple mechanisms, including mitochondrial dysfunction, excessive fatty acid oxidation, decreased activity of antioxidant enzymes (glutathione peroxidase, superoxide dismutase, catalase), and reduced intracellular levels of glutathione, vitamin C, and vitamin E [1]. In animal models of Mg deficiency, plasma malondialdehyde, a marker of lipid peroxidation, is markedly increased, while total antioxidant capacity is reduced, accompanied by hypertriglyceridemia and decreased HDL cholesterol [27]. The resulting oxidative modification of LDL cholesterol is a critical atherogenic stimulus, promoting foam cell formation and atherosclerotic plaque development [28,29].

Importantly, Mg deficiency induces a systemic low-grade inflammatory state mediated in part by substance P (SP), a neuropeptide tachykinin. Experimental data demonstrate that dietary Mg deficiency in rodents causes an early and dramatic increase in serum SP (detectable within 5 days), preceding the subsequent elevation of IL-1, IL-6, and TNF-α by approximately 2 weeks [30]. This “neurogenic inflammation” hypothesis proposes that Mg deficiency lowers the threshold for SP release from sensory neurons, triggering downstream cytokine cascades and mast cell degranulation that sustain both vascular and systemic inflammation [30]. The convergence of neurogenic and vascular inflammation under conditions of Mg deficiency is particularly relevant to the theme of this review, as it provides a mechanistic bridge between vascular endotheliopathy and pain sensitization.

## 4. Magnesium, Peripheral Arterial Disease, and Ischemic Pain

### 4.1. Epidemiological Evidence Linking Magnesium to Peripheral Arterial Disease

Peripheral arterial disease, defined by reduced ankle-brachial index (<0.9) reflecting atherosclerotic obstruction of lower limb arteries, causes ischemic pain ranging from intermittent claudication to rest pain and critical limb ischemia [31]. Clinically, ischemic pain in PAD exists along a spectrum, from exertional claudication caused by demand–supply mismatch during walking to rest pain associated with severe perfusion failure. In advanced disease, persistent ischemia may sensitize peripheral nociceptors and coexist with neuropathic symptoms, particularly in patients with diabetes or microvascular dysfunction. Several large epidemiological cohorts have established an independent inverse association between Mg status and PAD incidence. In the Atherosclerosis Risk in Communities (ARIC) study, which followed 11,839 participants over a median of 23 years, those in the lowest quartile of serum Mg (<1.5 mEq/L) had a hazard ratio for incident PAD of 1.96 (95% CI: 1.40–2.74) compared with those in the highest quartile [26].

In a cross-sectional analysis of 5969 NHANES participants (1999–2004), dietary Mg intake was significantly and negatively associated with PAD after adjustment for all relevant covariates, with individuals in the lowest quartile of Mg intake having an OR of 1.56 (95% CI: 1.02–2.39) for PAD compared with the highest quartile [32]. Among diabetic patients with PAD of the lower limbs, serum Mg levels were significantly lower (1.36 ± 0.6 mg/dL) than in diabetic patients without PAD (1.68 ± 0.4 mg/dL) and healthy controls (2.2 ± 0.4 mg/dL), with low Mg correlating with both arterial Doppler pathology and glycated hemoglobin [33]. Critically, a prospective study of 323 patients with symptomatic PAD and intermittent claudication demonstrated that low serum Mg (<0.76 mmol/L) was associated with a 3.29-fold increased risk of neurological events, including ischemic stroke, over a 20-month follow-up period, underscoring the systemic neurovascular consequences of hypomagnesemia in atherosclerotic disease [34].

### 4.2. Mechanisms of Ischemic Pain in the Context of Magnesium Deficiency

The ischemic pain of PAD arises from a complex interplay of tissue hypoxia, inflammatory mediator accumulation, and peripheral sensitization of nociceptors. In states of Mg deficiency, several mechanisms amplify this ischemic pain phenotype. First, impaired endothelial NO production and elevated ET-1 reduce vasodilatory reserve in the microcirculation, worsening tissue ischemia distal to stenotic lesions beyond what the macrovascular disease alone would predict [1]. Second, the pro-inflammatory vascular milieu created by Mg deficiency, with elevated cytokines, VCAM-1, and platelet aggregation, accelerates thrombus formation and plaque progression, reducing perfusion further [28,35]. Third, Mg deficiency augments the production of thromboxane A_2_, a potent vasoconstrictor and platelet-aggregating agent, and reduces the protective effects of PGI_2_, shifting the prostanoid balance toward vasoconstriction and platelet activation [35].

At the nociceptor level, ischemia generates metabolic byproducts including protons, lactate, bradykinin, ATP, and prostaglandins that activate acid-sensing ion channels (ASICs), TRPV1, and purinergic receptors on peripheral nociceptors. The inflammatory microenvironment created by Mg deficiency, with elevated substance P, histamine, and cytokines, further sensitizes these receptors, lowering the activation threshold for ischemic pain [30]. Additionally, Mg deficiency, by reducing intracellular Mg concentrations in sensory neurons, may facilitate calcium influx through voltage-gated channels, contributing to peripheral sensitization of C and Aδ nociceptors [36].

The diabetic milieu represents a particularly severe intersection of Mg deficiency and ischemic vulnerability. In patients with type 2 diabetes and PAD, both macrovascular atherosclerosis and microvascular dysfunction coexist, and hypomagnesemia correlates with the severity of both [33]. Microvascular disease in diabetes, manifested as retinopathy, nephropathy, and peripheral neuropathy, is aggravated by oxidative stress, glycation end-product accumulation, and endothelial injury, which are all amplified by Mg deficiency [37]. The net effect is a clinical phenotype in which ischemic limb pain and neuropathic pain frequently coexist and mutually reinforce each other.

## 5. Magnesium and Neuropathic Pain: Neurochemical and Neuromodulatory Mechanisms

### 5.1. NMDA Receptor Antagonism and Central Sensitization

Neuropathic pain is generally understood as pain arising from a lesion or disease of the somatosensory nervous system and involves ectopic neuronal activity, peripheral sensitization, central sensitization, and neuroimmune interactions [38]. Within this framework, Mg is particularly relevant because of its voltage-dependent modulation of the N-methyl-D-aspartate (NMDA) receptor [36]. The primary mechanism by which Mg exerts antinociceptive effects in neuropathic pain states is through voltage-dependent blockade of the NMDA receptor, a ligand-gated ion channel that plays a central role in synaptic plasticity and the initiation and maintenance of central sensitization [36]. Under physiological conditions, Mg^2+^ ions occupy the channel pore of NMDA receptors in a voltage-dependent manner at resting membrane potentials (approximately −70 mV), preventing calcium influx even when both glycine and glutamate, the two co-agonists of NMDA receptors, are simultaneously present [36]. This Mg block is the principal mechanism preventing tonic NMDA receptor activation and limiting calcium-mediated neuronal excitability under basal conditions.

In the context of sustained peripheral nociceptive input, as occurs in ischemic or neuropathic pain states, the repetitive depolarization of dorsal horn neurons releases the Mg block from NMDA receptors, enabling calcium influx and the induction of central sensitization [36]. Central sensitization is a state of enhanced excitability and synaptic efficacy within central nociceptive pathways, encompassing wind-up, long-term potentiation of synaptic strength, reduced pain threshold, allodynia, and hyperalgesia [39,40]. Magnesium deficiency exacerbates this process by reducing the efficacy of the Mg block at NMDA receptors, effectively lowering the threshold for calcium-mediated central sensitization to occur at any given membrane potential [41].

**Figure 3:**
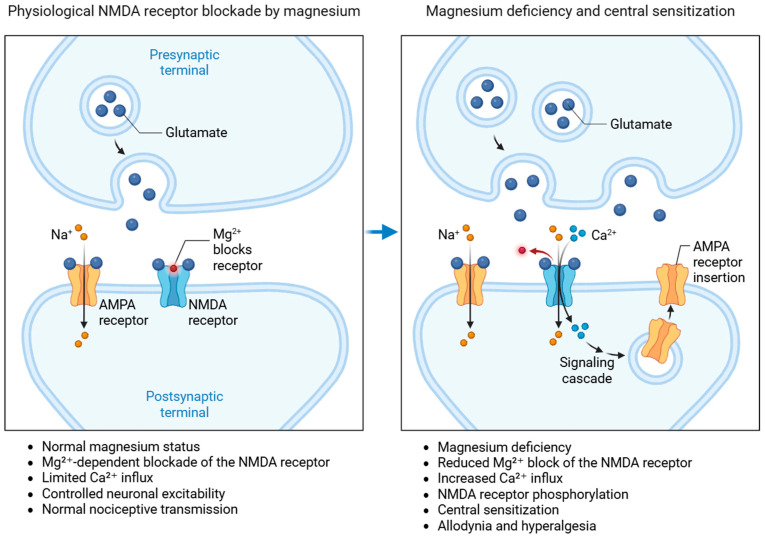
Schematic comparison of physiological NMDA receptor regulation by magnesium and enhanced excitatory signaling under magnesium deficiency. Under normal magnesium status, Mg^2+^ exerts a voltage-dependent blockade of the NMDA receptor, limiting Ca^2+^ influx, maintaining controlled neuronal excitability, and supporting normal nociceptive transmission. In contrast, magnesium deficiency reduces Mg^2+^-dependent blockade of the NMDA receptor, resulting in increased Ca^2+^ influx, activation of intracellular signaling cascades, NMDA receptor phosphorylation, and AMPA receptor insertion into the postsynaptic membrane. These changes promote central sensitization and contribute to pain amplification, manifested clinically as allodynia and hyperalgesia. Arrows indicate the proposed direction of excitatory signaling and downstream sensitization processes; they are schematic and do not represent quantified changes in molecular activity or clinical effect size.

In a landmark animal study, Mg supplementation in streptozocin-induced diabetic rats, a model of diabetic neuropathic pain, abolished thermal and tactile allodynia and delayed the development of mechanical hypersensitivity [42]. Critically, this protective effect was associated with the prevention of increased phosphorylation of the NMDA receptor NR1 subunit in the spinal cord dorsal horn, a molecular marker of central sensitization, demonstrating that Mg acts directly to modulate spinal nociceptive processing via the NMDA receptor pathway [42]. Complementing this, cerebrospinal fluid Mg concentrations are significantly lower in neuropathic rats compared with control animals, suggesting that spinal Mg depletion is both a consequence and a permissive factor for neuropathic pain chronification [41]. The proposed mechanism by which magnesium deficiency facilitates NMDA receptor dysregulation and central sensitization is illustrated in Figure 3.

### 5.2. Neuroinflammation, Microglia, and Glial-Mediated Pain Amplification

Most evidence discussed in this subsection derives from preclinical and in vitro studies; therefore, these mechanisms should be interpreted as biologically plausible pathways rather than as clinically established therapeutic effects.

Beyond the NMDA receptor, Mg modulates neuropathic pain through its anti-neuroinflammatory actions. Neuroinflammation, characterized by microglial activation, astrocyte reactivity, cytokine release, and prostaglandin synthesis, plays a critical role in initiating and perpetuating chronic pain by amplifying nociceptive transmission in the dorsal horn [38,43]. Mg deficiency in the brain upregulates neuroinflammation-associated gene expression in the hippocampus and cortex, activates microglia, and increases the production of pro-inflammatory cytokines including TNF-α, IL-1α, IL-1β, and IL-6 [43].

Mechanistically, low extracellular Mg reduces the voltage-dependent Mg block of NMDA receptors on microglial cells, enabling calcium influx and triggering the release of ROS, cytokines, and prostaglandin E_2_ (PGE_2_), all of which sensitize adjacent nociceptive neurons and maintain the neuroinflammatory cycle [43]. Mg inhibits the activation of NF-κB in primary microglia exposed to lipopolysaccharide, suppresses the release of TNF-α, IL-1α, IL-1β, and IL-6, and promotes a shift in microglial polarization toward the anti-inflammatory M2 phenotype. Mg also attenuates calcium entry through purinergic channels in microglia, limiting the acquisition of a neurotoxic phenotype.

Substance P (SP), as noted above, is an early effector of neurogenic inflammation induced by Mg deficiency. In the central nervous system, SP is released from sensory neurons and acts on neurokinin 1 receptors (NK1-R) expressed by neurons, astrocytes, and microglia. SP activates microglia to release cytokines, PGE_2_, NO, and ROS, amplifying neuroinflammatory signaling. Importantly, low Mg inhibits neprilysin, the principal SP-degrading enzyme, resulting in SP accumulation that precedes and drives subsequent inflammatory cascades. This Mg deficiency–SP–cytokine axis represents a neurochemical pathway through which systemic Mg status can directly modulate central pain processing.

### 5.3. Blood–Brain Barrier Integrity and Neurovascular Pain Coupling

Evidence linking magnesium to blood–brain barrier integrity is derived mainly from animal models of brain injury or ischemia and in vitro BBB models. Therefore, the relevance of these mechanisms to chronic neuropathic pain in humans remains translational and requires further clinical validation.

The blood–brain barrier (BBB) represents a critical interface between the peripheral vasculature and the central nervous system, and its structural and functional integrity shapes the neuroimmune environment of pain-processing centers [43,44]. Preclinical evidence suggests that magnesium may help preserve BBB integrity by attenuating excitotoxic, inflammatory, oxidative, and edema-related pathways rather than through a single disease-specific mechanism. In a randomized experimental rat model of intraperitoneal sepsis, magnesium sulfate administration attenuated Evans blue extravasation, a marker of increased BBB permeability, and reduced brain edema formation compared with untreated septic animals [45]. Similar BBB-protective effects have also been described in experimental CNS injury models, but these findings should be interpreted as preclinical evidence and should not be extrapolated directly to chronic neuropathic pain populations. Overall, adequate magnesium status may be relevant to central neurovascular integrity, but its clinical significance for human pain modulation remains unproven.

### 5.4. Diabetic Peripheral Neuropathy: A Model of Convergent Pathology

Diabetic peripheral neuropathy (DPN) represents a particularly compelling model in which Mg deficiency, microvascular disease, and neuropathic pain converge. DPN complicates 8–16% of diabetes mellitus cases and is characterized by aberrant symptoms of spontaneous and stimulus-evoked pain including burning pain, allodynia, and hyperalgesia, attributable to structural and functional damage to peripheral sensory neurons and their supporting vasculature [42]. Mg deficiency is more prevalent and more severe in diabetic patients with peripheral neuropathy than in those without this complication. Intracellular Mg levels in diabetic patients with peripheral neuropathy are significantly lower than in those without this complication (1.2 ± 0.5 vs. 1.5 ± 0.6 μg/mg protein) [46].

Clinical studies have demonstrated that lower serum Mg is significantly associated with electromyographic signs of polyneuropathy in diabetic patients, and that oral Mg supplementation can improve nerve conduction velocity in Mg-depleted type 1 diabetic patients, particularly in younger individuals with shorter disease duration [47]. A cross-sectional study of 116 type 2 diabetic patients found that mean Mg levels were significantly lower in those with confirmed polyneuropathy on electromyography (ENMG) than in those without, and patients with additional complications beyond polyneuropathy had the lowest Mg levels [48]. A further study demonstrated that 56% of diabetic patients had hypomagnesemia, with the proportion rising in those with concurrent peripheral neuropathy [49].

The pathophysiology of DPN is closely linked to endoneurial microvascular disease, in which inflammation, basement membrane thickening, impaired perfusion, and hypoxia contribute to neural injury [37,50]. Mg deficiency amplifies this injury through increased oxidative stress, elevated vascular endothelin-1, reduced NO bioavailability, and accelerated glycation end-product accumulation. Concurrently, reduced intracellular Mg in peripheral neurons reduces the voltage-dependent NMDA block in dorsal root ganglion neurons, facilitating ectopic discharge and peripheral sensitization [36]. In experimental models, oral Mg supplementation prevented allodynia and thermal hyperalgesia in diabetic rats, supporting the relevance of the NMDA-mediated pathway discussed above [42].

## 6. Magnesium, Metabolic Dysregulation, and the Neurovascular-Metabolic Interface

### 6.1. Insulin Resistance, Type 2 Diabetes, and Magnesium

The bidirectional relationship between Mg and insulin signaling creates a pathological loop of particular relevance to neurovascular pain. Intracellular Mg is required for the autophosphorylation of the insulin receptor’s tyrosine kinase domain; reduced intracellular Mg impairs downstream insulin signaling and cellular glucose uptake, directly contributing to insulin resistance [11,51]. Conversely, insulin and glucose regulate Mg homeostasis; insulin normally stimulates cellular Mg uptake, and in insulin-resistant or insulin-deficient states, this stimulus is blunted, leading to progressive intracellular Mg depletion [52,53]. In patients with type 2 diabetes, the insulin-stimulated decrement in plasma Mg is significantly smaller than in non-diabetic controls, confirming impaired insulin-mediated Mg transport as a consequence of insulin resistance [53].

In addition to impaired cellular Mg uptake, glycosuria, a hallmark of poorly controlled diabetes, causes osmotic diuresis that increases urinary Mg excretion, further depleting systemic Mg stores [12]. The combination of reduced dietary intake, impaired intestinal absorption, and increased renal wasting explains why hypomagnesemia is present in 25–38% of unselected patients with type 2 diabetes, with higher prevalence in those with poorly controlled glycemia and complications [46]. This chronic Mg insufficiency, operating in the context of hyperglycemia and dyslipidemia, amplifies oxidative stress, vascular endothelial damage, and neuroinflammation, creating an environment in which ischemic and neuropathic pain phenotypes can develop and progress.

### 6.2. Magnesium and the Metabolic Syndrome

Magnesium deficiency is not isolated to diabetes but is broadly associated with the metabolic syndrome, a constellation of central obesity, hypertriglyceridemia, low HDL, hypertension, and impaired fasting glucose, each of which independently predisposes to cardiovascular and neuropathic disease [54]. Mg supplementation ameliorates multiple components of the metabolic syndrome: it improves insulin sensitivity, reduces serum triglycerides, increases HDL cholesterol, attenuates inflammatory biomarkers (CRP, IL-6, fibrinogen), and improves endothelium-dependent vasodilation [54,55]. A dose–response meta-analysis of 40 prospective cohort studies (>1 million participants, follow-up 4–30 years) demonstrated that each 100 mg/day increment in dietary Mg was associated with a 22% reduction in the risk of heart failure, a 7% reduction in stroke risk, a 19% reduction in type 2 diabetes risk, and a 10% reduction in all-cause mortality [56]. However, these findings should be interpreted cautiously because prospective dietary studies remain vulnerable to dietary measurement error, residual confounding, and reverse causality. Therefore, randomized controlled trials with baseline magnesium stratification are needed to determine whether magnesium repletion causally reduces cardiometabolic and neurovascular risk.

These associations are particularly relevant in the context of neurovascular pain because metabolic syndrome components, especially hypertension and hyperglycemia, directly damage both large and small vessel endothelium, creating the microvascular substrate for ischemic and neuropathic pain. The extent to which Mg repletion might attenuate this risk at the individual patient level remains an open clinical question warranting larger randomized trials [55].

## 7. Clinical Evidence: Magnesium in Pain Management

Across the clinical studies discussed below, cross-study comparisons are limited by substantial heterogeneity in magnesium formulation, elemental magnesium dose, route of administration, treatment duration, and baseline magnesium status. Available studies have used oral elemental magnesium, magnesium oxide, intravenous magnesium sulfate, and different dosing schedules, making it inappropriate to assume equivalent bioavailability or clinical effects across trials.

### 7.1. Neuropathic Pain

Clinical evidence for Mg in neuropathic pain management is suggestive but heterogeneous, reflecting the diversity of neuropathic pain etiologies, administration routes, dosages, and outcome measures employed across trials [57]. Importantly, magnesium should not be interpreted as an established first-line pharmacotherapy for neuropathic pain, but rather as a biologically plausible adjunctive strategy requiring further controlled trials [57,58]. A systematic review identified nine randomized controlled trials (418 participants) evaluating Mg in chronic pain conditions, including neuropathic pain, migraine, complex regional pain syndrome, and low back pain with neuropathic features [57]. Heterogeneity across studies precluded meta-analysis, but efficacy signals were observed in several trials, particularly with intravenous Mg administration in conditions characterized by central sensitization [57]. Notably, in the randomized trial by Pickering et al., oral magnesium treatment did not show significant superiority over placebo after 4 weeks in patients with neuropathic pain, although exploratory changes in pain paroxysms and emotional impact were reported [59]. This negative finding weakens any direct efficacy claim for oral magnesium in neuropathic pain and supports interpreting magnesium as a biologically plausible adjunctive strategy rather than an established analgesic therapy.

Intravenous Mg sulfate administered weekly to patients with postherpetic neuralgia or causalgia, both paradigmatic neuropathic pain conditions, produced a ≥3-point reduction in VAS pain scores in four of eight treated patients, with no serious adverse events [60]. In patients with low back pain with a neuropathic component, a double-blind trial of sequential intravenous followed by oral Mg therapy demonstrated significant improvements in pain intensity and lumbar range of motion [36]. For diabetic peripheral neuropathy specifically, oral Mg supplementation has been explored in small studies, and the therapeutic rationale remains biologically plausible given the links between Mg deficiency, NMDA receptor sensitization, and diabetes-related nerve injury [36].

Several small studies and case-based reports have explored magnesium in specific neuropathic pain conditions such as postherpetic neuralgia and chemotherapy-induced peripheral neuropathy, but the evidence remains heterogeneous and insufficient for definitive clinical recommendations [36,57].

### 7.2. Ischemic Pain and Cardiovascular Outcomes

The clinical evidence linking Mg to ischemic pain reduction is more indirect, operating through improvement in endothelial function, reduction in coronary vasospasm, and improved exercise tolerance. In the RCT by Shechter et al., stable CAD patients receiving oral Mg supplementation for 6 months showed significantly better exercise tolerance (9.3 vs. 7.3 min on the Bruce protocol, *p* = 0.05) and fewer ischemic ST-segment changes (4 vs. 10 patients, *p* = 0.05) compared with placebo; these results are compatible with improved myocardial perfusion and reduced ischemic burden [19]. These findings suggest that Mg supplementation may influence ischemic burden through endothelial and microvascular mechanisms, although direct evidence for ischemia-mediated limb pain relief remains limited.

In diabetic patients with foot ulcers, an extreme manifestation of ischemic and neuropathic convergence, a 12-week randomized trial of Mg supplementation (250 mg/day) produced significantly greater reductions in ulcer dimensions, fasting plasma glucose, HbA1c, serum CRP, and improvements in insulin sensitivity and total antioxidant capacity compared with placebo [61]. Although not a pain-specific endpoint trial, these results demonstrate that Mg supplementation produces measurable improvements in metabolic and inflammatory parameters directly relevant to the tissue microenvironment driving neuropathic and ischemic pain in diabetic foot disease. Nevertheless, no adequately powered clinical trial has specifically evaluated Mg supplementation for PAD-related claudication or ischemic limb pain as a primary endpoint. Because the available clinical evidence includes both direct pain outcomes and indirect vascular or metabolic endpoints, the relevant clinical and translational studies are summarized in Table 2.

## 8. Integrative Framework: Magnesium as a Neurovascular-Metabolic Modulator

The totality of the evidence reviewed herein supports a conceptual reframing of Mg beyond its role as a simple analgesic supplement. Mg operates as a neurovascular–metabolic modulator whose adequate status is necessary to maintain the integrity of each component of the neurovascular unit: the endothelium, the smooth muscle cell, the basement membrane, the perivascular nerve, and the central pain-processing circuitry [1,6,43,44].

In patients with atherosclerosis and its metabolic comorbidities, Mg deficiency should not be interpreted merely as an epiphenomenon; mechanistic evidence suggests that it may participate in several interrelated vascular, metabolic, and neural pathways. It may destabilize endothelial homeostasis through oxidative stress and NF-κB activation, promote vascular calcification through VSMC osteogenic transdifferentiation, impair insulin signaling through defective tyrosine kinase activity, sensitize peripheral nociceptors through calcium channel dysregulation, and lower the threshold for central sensitization through reduced NMDA receptor Mg block [11,15,24,36,39]. These processes do not operate in isolation; they form a pathophysiological network in which vascular injury and pain sensitization mutually reinforce each other.

As summarized in Figure 1, chronic magnesium deficiency may act as an upstream driver of vascular dysfunction, neural sensitization, and metabolic dysregulation. These pathways may converge clinically in conditions such as diabetic peripheral neuropathy with PAD, where impaired endoneurial microcirculation can promote neural ischemia while reduced spinal Mg^2+^ availability may facilitate NMDA receptor-mediated amplification of nociceptive signaling [33,42,46]. This convergence provides a mechanistic explanation for the frequent coexistence and mutual reinforcement of ischemic and neuropathic pain phenotypes in metabolically vulnerable patients.

## 9. Clinical Implications and Therapeutic Considerations

Several practical implications emerge from this framework, although these considerations are intended to guide interpretation of the literature and future trial design rather than to provide disease-specific treatment recommendations. First, Mg status assessment may be considered as part of the metabolic evaluation of patients with established atherosclerosis, PAD, or chronic neuropathic pain, particularly in those with diabetes, CKD, or nutritional risk factors for deficiency [3,9,12].

Second, Mg supplementation strategies should be differentiated by clinical context. In patients with chronic neuropathic pain and documented hypomagnesemia, oral supplementation targeting repletion of Mg stores may represent a biologically plausible adjunctive strategy, particularly given its favorable safety profile and low cost [57,59]. In acute settings such as perioperative neuroprotection or management of refractory neuropathic pain, intravenous Mg administration may offer more reliable plasma Mg elevation and more rapid NMDA receptor effects [36].

Third, magnesium dosing and formulation should be interpreted carefully in both clinical practice and research. Dosing should be reported as elemental magnesium rather than the total weight of the magnesium salt. The recommended dietary allowance for magnesium in adults generally ranges from 310 to 420 mg/day depending on age and sex, whereas the tolerable upper intake level for supplemental magnesium in adults is 350 mg/day; this upper limit applies only to magnesium from supplements or medications, not to magnesium naturally present in foods. Dietary magnesium absorption is typically estimated at approximately 30–40%, and the bioavailability of supplemental magnesium varies by formulation. More soluble magnesium salts, including citrate, lactate, chloride, and aspartate, generally show greater bioavailability than poorly soluble inorganic forms such as magnesium oxide, although comparative absorption varies across study designs and populations [3,62,63,64]. Magnesium L-threonate has been proposed to have greater central nervous system relevance based largely on preclinical and early translational data, but its clinical superiority for neuropathic pain or neurovascular endpoints remains unproven [43]. Therefore, future trials should clearly report elemental magnesium dose, formulation, route of administration, duration, baseline magnesium status, renal function, and adverse gastrointestinal effects. A broader supplementation framework should also consider dietary magnesium intake, formulation-dependent bioavailability, gastrointestinal tolerability, renal function, medication use, and interactions with other nutrients such as calcium, vitamin D, potassium, and dietary fiber [7].

Fourth, the timing and duration of Mg intervention are important variables that remain underexplored. Given that chronic Mg deficiency requires sustained correction rather than acute loading to reverse intracellular depletion, clinical trials should incorporate sufficiently long follow-up durations, typically at least 3–6 months, and should measure both intracellular and serum Mg to confirm adequate repletion [46,59].

## 10. Limitations and Future Research Directions

Several important limitations constrain the current evidence base. Many of the mechanistic insights derive from in vitro cell culture studies or animal models that may not fully recapitulate human physiology, particularly the complex interaction between systemic hypomagnesemia, multimorbid vascular disease, and chronic pain in aging patients [21,57]. Clinical trials of Mg in neuropathic pain are generally small, heterogeneous in their patient populations and Mg formulations, and limited in follow-up duration, precluding definitive conclusions about efficacy or optimal dosing strategies [57,59]. Accordingly, the mechanistic findings summarized in this review should be interpreted as hypothesis-generating and should not be directly extrapolated to clinical efficacy without adequately powered human trials using pain-specific endpoints.

The absence of a validated, widely available biomarker for intracellular Mg status in routine clinical practice remains a significant barrier to research and clinical translation. Future studies should prioritize the validation of accessible Mg biomarkers that reflect functional intracellular Mg status. Additionally, the potential interaction between Mg and other nutritional cofactors, including vitamin D, potassium, and calcium, warrants further exploration in the context of cardiovascular and neuropathic pain [43].

Future research should also address the following specific gaps: (1) prospective randomized controlled trials evaluating Mg supplementation on patient-reported pain outcomes in PAD with claudication; (2) mechanistic studies elucidating the relative contributions of peripheral vs. central Mg deficiency to neuropathic pain phenotypes; (3) investigation of Mg status as a modifier of analgesic responsiveness in patients with chronic neuropathic pain; (4) evaluation of whether Mg repletion reduces the incidence of chronic post-surgical pain in vascular surgical patients; and (5) pharmacokinetic studies comparing BBB penetration of different Mg formulations in patients with neurological pain conditions.

## 11. Conclusions

Magnesium occupies a unique position at the intersection of vascular biology, metabolic regulation, and pain neuroscience. This narrative review has outlined the evidence supporting a reconceptualization of Mg not merely as an analgesic supplement, but as a neurovascular–metabolic modulator whose chronic low magnesium status is associated with endothelial dysfunction, atherosclerotic burden, impaired microcirculatory function, and sensitization of peripheral and central pain pathways. Mechanistic studies suggest that magnesium deficiency may contribute to these processes, but direct causal evidence in human ischemic and neuropathic pain populations remains limited. In clinically vulnerable populations, including patients with atherosclerosis, type 2 diabetes, CKD, or chronic nutritional insufficiency, hypomagnesemia may. function as a common pathophysiological denominator that links vascular injury to the development and perpetuation of ischemic and neuropathic pain phenotypes.

The recognition of this integrative role for Mg has both scientific and therapeutic implications. Scientifically, it motivates the development of research frameworks that examine vascular and neuropathic disease as mechanistically connected through shared metabolic and inflammatory pathways. Therapeutically, it supports consideration of Mg status assessment in patients with chronic pain in the context of vascular or metabolic disease, particularly when nutritional deficiency or hypomagnesemia is suspected. From a nutritional perspective, achieving and maintaining adequate Mg status through dietary sources such as whole grains, leafy greens, legumes, and nuts, with supplementation considered when clinically appropriate, represents a biologically rational strategy for addressing an underappreciated contributor to neurovascular vulnerability.

## Figures and Tables

**Table 1:** Clinical assessment methods and contextual risk factors for magnesium deficiency. Serum total magnesium (Mg) is widely available but may poorly reflect intracellular Mg status. Erythrocyte Mg, ionized Mg, and 24 h urinary Mg may provide additional information, although their clinical availability varies. Clinical risk factors should be considered when interpreting Mg status, particularly in patients with diabetes, chronic kidney disease (CKD), proton pump inhibitor (PPI) use, loop diuretic use, or poor dietary intake. RBC Mg should be interpreted cautiously because erythrocytes lack nuclei and mitochondria and may not fully reflect Mg content in nucleated or metabolically active cells. The serum Ca/Mg ratio is included as an emerging complementary index rather than a validated standalone diagnostic marker.

Assessment Method	Clinical Characteristics
Serum total Mg	Easy and widely available, but a poor marker of intracellular Mg
RBC Mg	Potential intracellular estimate, but limited clinical availability; interpretation should be cautious because erythrocytes lack nuclei and mitochondria and may not fully represent Mg content or concentration in nucleated cells or metabolically active tissues
Ionized Mg	Physiologically relevant, mainly used in research settings
24 h urinary Mg	Useful for evaluating renal Mg wasting
Clinical risk factors	Diabetes, CKD, PPI use, loop diuretic use, poor dietary intake
Serum Ca/Mg ratio	Emerging complementary index of Mg–Ca balance; may help identify adverse Mg/Ca imbalance, but requires further validation and should not replace conventional Mg assessment

**Table 2:** Clinical and translational evidence for magnesium in neuropathic and ischemia-related pain conditions. Available evidence includes preclinical studies, observational associations, small clinical series, randomized clinical trials, and systematic reviews. The evidence for neuropathic pain remains heterogeneous, whereas the evidence for PAD-related ischemic limb pain is mainly indirect, deriving from vascular, metabolic, or epidemiological endpoints rather than pain-specific clinical trials. Study design and evidence level are shown to clarify the strength and limitations of each source.

Study	Study Design/Evidence Level	Condition/Model	Mg-Related Intervention or Exposure	Key Finding	Interpretation
Park et al., 2020 [57]	Systematic review of RCTs/clinical evidence	Chronic pain, including neuropathic pain	Systematic review of Mg interventions	Evidence was equivocal and heterogeneous; some efficacy signals were reported	Overall clinical evidence for Mg in chronic pain
Pickering et al., 2011 [59]	Randomized clinical trial/direct clinical evidence	Neuropathic pain	Oral Mg treatment	No significant superiority over placebo after 4 weeks; exploratory changes in pain paroxysms/emotional impact	Direct but inconclusive clinical evidence
Tanaka et al., 1998 [60]	Small clinical series/low-level clinical evidence	Postherpetic neuralgia/causalgia	IV Mg sulfate	Pain reduction reported in a small clinical series	Supports possible Mg use in refractory neuropathic pain
Rondón et al., 2010 [42]	Animal study/preclinical evidence	Diabetic neuropathic pain model	Mg supplementation	Reduced hypersensitivity and spinal NMDA receptor phosphorylation	Mechanistic support for Mg in diabetic neuropathic pain
Mühürdaroğlu et al., 2021 [48]	Cross-sectional clinical study/associative evidence	Diabetic polyneuropathy	Serum Mg status	Lower Mg associated with confirmed polyneuropathy	Clinical association with diabetic neuropathy
Shechter et al., 2000 [19]	Randomized clinical trial/indirect vascular evidence	Stable coronary artery disease	Oral Mg supplementation	Improved FMD, exercise tolerance, and ischemic ST changes	Indirect evidence for ischemia-related symptoms
Mortazavi et al., 2013 [20]	Randomized clinical trial/indirect vascular evidence	Hemodialysis patients	MgO supplementation	Improved carotid intima-media thickness and flow-mediated dilatation	Indirect relevance to vascular dysfunction
Menez et al., 2020 [26]	Prospective cohort study/epidemiologic evidence	PAD	Serum Mg status	Low serum Mg associated with increased incident PAD risk	Supports Mg–PAD association
Wu et al., 2023 [32]	Cross-sectional NHANES analysis/epidemiologic evidence	PAD	Dietary Mg intake	Lower dietary Mg intake associated with PAD	Epidemiologic support for Mg–PAD relationship
Razzaghi et al., 2018 [61]	Randomized clinical trial/indirect metabolic-wound evidence	Diabetic foot ulcer	Oral Mg supplementation	Improved wound, glycemic, inflammatory, and antioxidant markers	Indirect relevance to diabetic ischemic-neuropathic tissue environment

## Data Availability

No new data were created or analyzed in this study. Data sharing is not applicable to this article.
